# [Retracted] Acetylsalicylic acid combined with diclofenac inhibits cartilage degradation in rabbit models of osteoarthritis

**DOI:** 10.3892/etm.2023.12199

**Published:** 2023-09-11

**Authors:** Jianqiang Liu, Changshun Wu, Dong Wang, Laicheng Wang, Shui Sun

Exp Ther Med 12:2177-2182, 2016; DOI: 10.3892/etm.2016.3560

Following the publication of this paper, it was drawn to the Editor’s attention by a concerned reader that, for the experiments showing hematoxylin and eosin staining of rabbit knee cartilage in Fig. 2 on p. 2179, there appeared to be a number of repeating patterns of tissue sections within the images themselves, calling into question the integrity and the authenticity of the presented data.

The Editorial Office have conducted an independent assessment of these data, and can confirm the veracity of the concerns that were raised by the reader; therefore, the Editor of *Experimental and Therapeutic Medicine* has decided that this paper should be retracted from the Journal on account of a lack of confidence in the presented data. The authors were asked for an explanation to account for these concerns, but the Editorial Office did not receive a reply. The Editor apologizes to the readership for any inconvenience caused.

